# Polymeric Coatings Based on Water-Soluble Trimethylammonium Copolymers for Antifouling Applications

**DOI:** 10.3390/molecules25071678

**Published:** 2020-04-06

**Authors:** Artemis Tsagdi, Denisa Druvari, Dionisios Panagiotaras, Pavlos Avramidis, Vlasoula Bekiari, Joannis K. Kallitsis

**Affiliations:** 1 Department of Chemistry, University of Patras, GR–26504 Patras, Greece; artemis.tsagdh@gmail.com (A.T.); druvari@upatras.gr (D.D.); 2 Department of Environment, Ionian University, M. Minotou-Giannopoulou 26, Zakynthos 29100, Greece; sakpanag@yahoo.gr; 3 Department of Geology, University of Patras, GR-26504 Patras, Greece; p.avramidis@upatras.gr; 4 Department of Animal Production, Fisheries and Aquaculture, University of Patras, 30200 Messolonghi, Greece; bbekiari@upatras.gr; 5 Foundation for Research and Technology-Hellas (FORTH)/Institute of Chemical Engineering Sciences (ICE-HT), Stadiou Str., Platani, P.O. Box 1414, GR-265 04 Rio-Patras, Greece

**Keywords:** water-soluble copolymers, crosslinked polymeric materials, quaternary trimethylammonium compounds, antifouling coatings, high-pressure cleaning, aquaculture nets

## Abstract

Crosslinked polymeric materials based on a quaternary trimethylammonium compound were developed and evaluated as potential antifouling coatings. For this purpose, two water-soluble random copolymers, poly(4-vinylbenzyltrimethylammonium chloride-co-acrylic acid) P(VBCTMAM-co-AAx) and poly(N,N-dimethylacrylamide-co-glycidylmethacrylate) P(DMAm-co-GMAx), were synthesized via free radical polymerization. A water based approach for the synthesis of P(VBCTMAM-co-AAx) copolymer was used. Coatings of the complementary reactive copolymers in different compositions were obtained by curing at 120 °C for one day and were used to coat aquaculture nets. These nets were evaluated in respect to their release rate using Total Organic Carbon (TOC) and Total Nitrogen (TN) measurements. Finally, the antifouling efficacy of these newly-composed durable coatings was investigated for 14 days in accelerated conditions. The results showed that this novel polymeric material provides contact-killing antifouling activity for a short time period, whereas it functions efficiently in biofouling removal after high-pressure cleaning.

## 1. Introduction

The development of biofouling in marine aquaculture applications represents a crucial financial and ecological problem. Any object immersed in the sea is rapidly colonized by a wide variety of organisms [1,2]. These bioaccumulations have adverse effects on surfaces submerged in the aquatic environment such as shipping vessels, aquaculture cages, offshore rigs and jetties [3]. In the case of aquaculture industry, biofouling has significant impacts on culture species, on farm infrastructure (immersed structures such as cages and netting) as well as on the natural ecosystem [4]. In order to combat this problem, the aquaculture industry needs to develop optimized biofouling management technologies.

An aquaculture farm usually consists of big cages which are netted with various synthetic materials. The development of biofouling on the nets, which blocks the mesh openings, is treated with periodical in situ cleaning of the fishing nets with high-pressure washers [5]. Nevertheless, this method is not cost effective, so a preventative approach is required that relies on using antifouling materials as coatings for the nets [6]. In the last few decades, the most common antifouling coatings were based on biocide-releasing paints of tributyl tin (TBT), copper and zinc-containing systems. Although very effective, these compounds raise serious issues to marine ecosystems, affecting both target and non-target organisms.

For this reason, the attention of research today is focused on the development of nontoxic metal-free polymeric antifouling (AF) and fouling release (FR) surfaces. The difference between the two types of coatings is that the first one prevents the attachment of biofoulants, whereas the latter weakens the adhesion of biofoulants to the surface, facilitating their removal by hydrodynamic stress during movement or mechanical cleaning [7]. AF coatings are usually prepared from hydrophilic polymers which have high-surface energies similar to water. Therefore, they perform well as AF agents because they prefer to remain in contact with water rather than an amphiphilic biomolecule like a protein. In contrast, FR coatings are mainly composed from very hydrophobic polymers with low energy that reduces the ability of biomolecules to interact strongly with the surface. The most widely used materials for FR coatings are based on polydimethylsiloxane (PDMS) and fluorinated materials [8,9,10,11]. However, due to the fact that these coatings suffer from several disadvantages, they are usually combined with hydrophilic components leading to amphiphilic surfaces with improved performance [12,13].

Several categories of hydrophilic surfaces are being explored for the design of successful AF coatings in marine environments. Hydrogels, polymeric materials that absorb a large amount of water, have been synthesized for antifouling purpose [14]. Due to their poor stability though, recent research is focused on charged networks, which offer a promising alternative for AF coatings. Among potential candidates, zwitterionic systems appear to be highly effective against marine fouling organisms [15,16,17], as well as anionic units [18,19] and cationic units based on quaternary ammonium compounds (QACs) with short alkyl chains [20,21,22,23,24] which are charged compounds that although less explored in the AF field showed encouraging results.

In our previous work, the reactive blending of copolymers with complementary reactive groups (AA and GMA) was applied to obtain self-standing antimicrobial membranes. Specifically, two series of copolymers poly (4-vinyl benzyl dimethylhexadecylammonium chloride-co-acrylic acid) P(VBCHAM-co-AAx) and poly (cetyltrimethylammonium 4-styrenesulfonate-co-glycidyl methacrylate) P(SSAmC_16_-co-GMAx) were combined in order to prepare crosslinked membranes, containing a quaternary N,N-dimethylhexadecylammonium group both covalently (VBCHAM) and electrostatically (SSAmC_16_) attached [25]. These membranes presented strong antimicrobial activity against S. aureus and P. Aeruginosa, while when applied as coatings on aquaculture nets exhibited high antifouling action as compared to the blank net, for a period of up to 35 days. This methodology was investigated in more detail in order to get a deeper understanding of the release behavior of these systems [9]. An interesting observation in both works was that the release rate in salt solution was maintained in much lower levels than in pure water, offering an additional advantage for potential antifouling applications in sea water.

Motivated by the above encouraging results, our aim in the present work is to investigate the role of chain length of the cationic group on antifouling activity by introducing trimethylamine instead of N,N-dimethylhexadecylamine into the P(VBC-co-AAx) copolymer. We demonstrated the quaternization reaction in a single green step using water as solvent. Taking advantage of the previously-mentioned crosslinking reaction between the acrylic acid and epoxide group, we prepared polymeric coatings of new water-soluble complementary copolymers P(VBCTMAM-co-AAx) and P(DMAm-co-GMAx). These coatings were studied for their antifouling efficiency on aquaculture nets in accelerated conditions. Thus, this study uses a simple and scalable fabrication approach for the development of hydrophilic, positively charged stable surfaces from water-soluble polymers as potential antifouling or fouling release paints.

## 2. Results and Discussion

The main idea of this work was to combine two water-soluble copolymers bearing complementary reactive groups together with positively charged ammonium groups in order to develop hydrophilic coatings stable in seawater. These materials will be further discussed in terms of their stability through release studies and of their antifouling activity in accelerated conditions.

### 2.1. Synthesis and Characterization of the P(VBCTMAM-co-AA20) Copolymer

**Scheme 1:**
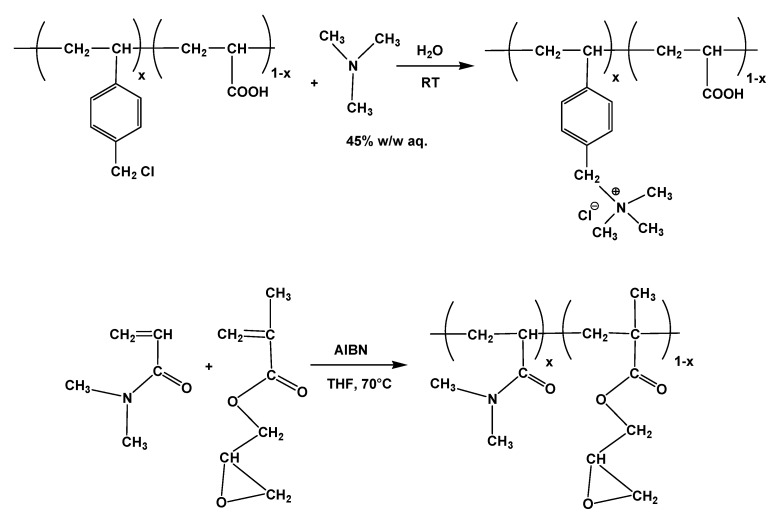
Synthesis of reactive copolymers P(VBCTMAM-co-AAx) and P(DMAm-co-GMAx).

**Figure 1:**
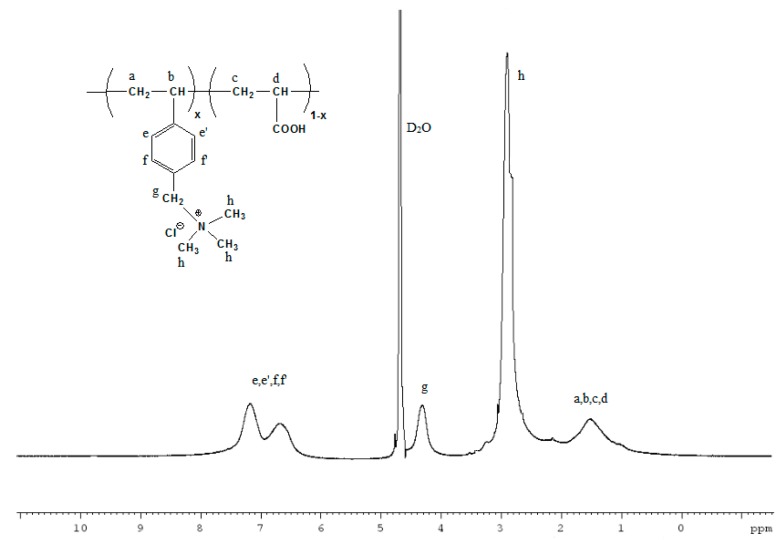
Proton Nuclear Magnetic Resonance (^1^H NMR) spectrum of the quaternized P(VBCTMAM-co-AA20) copolymer in D_2_O.

Precursor copolymers were synthesized via conventional free radical polymerization using AIBN as initiator (Scheme 1). The structure of the synthesized copolymers was confirmed by Proton Nuclear Magnetic Resonance (^1^H NMR) spectroscopy. The chemical composition of the synthesized copolymers was 80/20 *w*/*w*, based on the integral ratio of the broad peaks in the range 1.1–2.4 ppm which are assigned to backbone protons of VBC and AA units and the peak at 4.5 ppm attributed to the protons of chloromethyl groups of VBC. Subsequently, the introduction of trimethylamine into the benzyl chloride moiety happened in a facile route, using water as solvent. Firstly, the P(VBC-co-AA20) copolymer was dispersed in water and then the aqueous trimethylamine solution 45% (*w*/*w*) was added to the suspension and left to react at room temperature for one day. Finally, a homogenous opaque yellowish solution of the polymer was obtained as an evidence of the successful quaternization reaction. To the best of our knowledge, this is the first report of this reaction in water solution, while in other studies several organic solvents were used for the amination of PVBC with trimethylamine [26]. The N-quaternization reaction was verified via comparison of the ^1^H NMR spectra of the product and its precursor copolymer. In particular, in the ^1^H NMR spectrum of the quaternized copolymer (Figure 1), it is evident that the peak located at 4.5 ppm corresponding to the protons of CH_2_Cl groups disappeared, while a new one emerges at 4.3 ppm. This new peak is attributed to the CH_2_ protons (g) attached to the quaternary nitrogen of trimethylamine. In addition, the peaks at 6.7–7.2 ppm result from the aromatic protons (e,e’,f,f’) and the broad peaks at 0.8–1.8 ppm are assigned to the backbone protons of the copolymer (a,b,c,d). Finally, the peak at 2.9 ppm corresponds to the three methyl groups of the quaternary ammonium moiety. The acidic protons of the acrylic acid units do not appear in the spectrum and this is partially attributed to the formation of the trimethylammonium salt with the excess of trimethylamine and also to the exchange of the acidic protons since they do not appear in the case of the precursor copolymer P(VBC-co-AA20) neither.

### 2.2. Synthesis and Characterization of Copolymer P(DMAm-co-GMA30)

**Figure 2:**
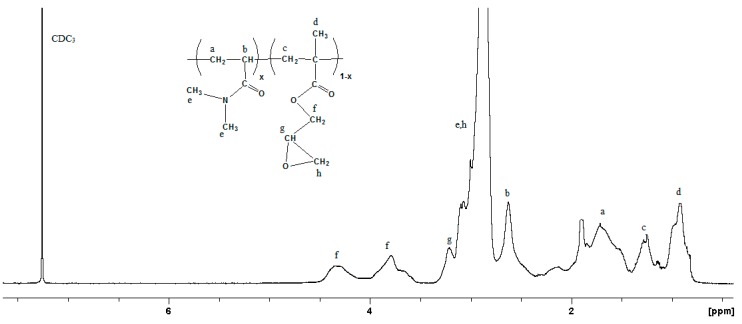
^1^H NMR spectrum of the P(DMAm-co-GMA30) copolymer in CDCl_3_.

The synthesis of the P(DMAm-co-GMAx) copolymer (Scheme 1) was conducted through free radical polymerization, as reported elsewhere [27]. The chemical composition of the synthesized copolymer was estimated through ^1^H NMR spectroscopy. Figure 2 shows the ^1^H NMR spectrum of the P(DMAm-co-GMAx) copolymer in D_2_O with 30 mol% GMA content. Concerning GMA, the peak at 2.9 ppm corresponds to the methylene protons (h) of the epoxy ring, whereas the peak at 3.2 ppm and the broad peaks at 3.8 and 4.4 ppm correspond to the methine proton (g) of the epoxy group and the -OCH_2_ protons (f) of GMA units, respectively. The α methyl group of GMA (d) appears at 0.9 ppm. The DMAm unit was confirmed by the broad peak at 2.7–3.2 ppm assigned to the methyl protons (e) of the amide.

### 2.3. Preparation of Antifouling Coatings

**Scheme 2:**
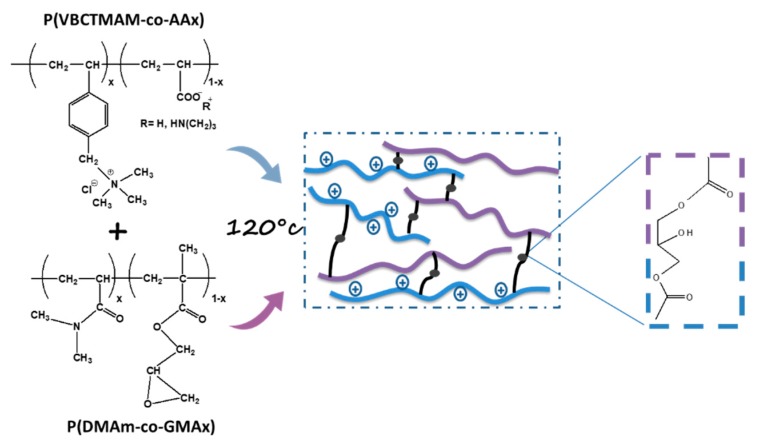
Crosslinking reaction between the P(VBCTMAM-co-AA20) and P(DMAm-co-GMA30).

The main goal of this study is the production of durable hydrophilic coatings for antifouling seawater applications. Taking advantage of our previous knowledge on the cross-linking reaction of the carboxyl group with the epoxy group after heat treatment at 120 °C [9,25], the two complementary copolymers P(VBCTMAM-co-AA20)/P(DMAm-co-GMA30) were blended in water and cured at 120 °C for one day in order to form crosslinked polymeric coatings (Scheme 2). Since the nets that were used in this work are composed from Nylon, such conditions can be easily applied.

The effect of the use of an excess of trimethylamine for the preparation of P(VBCTMAM-co-AA20) copolymer resulting in the formation of the trimethylammonium salt of the acrylic acid moieties was also investigated. Thus, copolymers synthesized with stoichiometric amount of amine were also tested and in all cases the crosslinking reaction was efficient. This was attributed to the fact that the ammonium salt of the acrylic acid reacted with the epoxides [28].

In an attempt to optimize the stability of the coatings, different compositions of the two complementary copolymers were investigated, while the content of the reactive units AA and GMA remained 20 and 30 mol%, respectively. The compositions were suitably chosen so that the obtained mixtures were rich in ammonium compounds, which were expected to provide the antifouling activity. Specifically, the content of the P(VBCTMAM-co-AA20) copolymer in the mixtures was 60%, 70% and 80% *w*/*w*.

**Figure 3:**
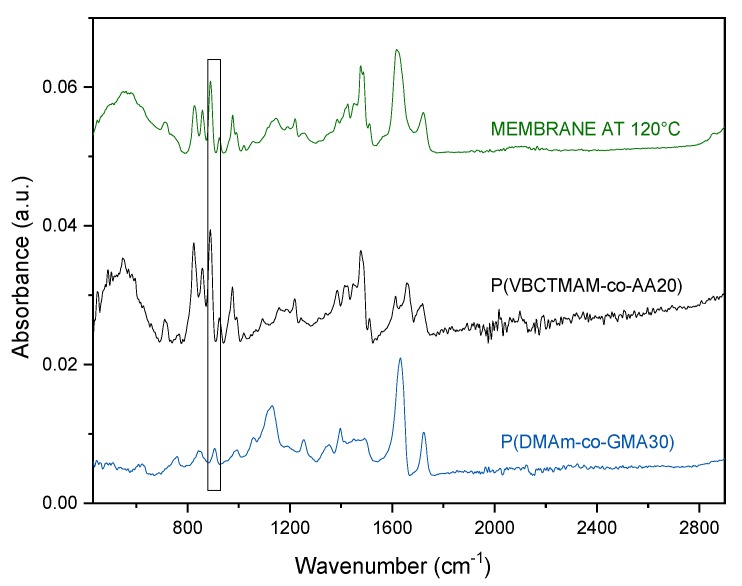
Attenuated Total Reflection Fourier Transform Infrared Spectroscopy (ATR-FTIR) spectra of the complementary copolymers and the crosslinked membrane P(VBCTMAM-co-AA20)/P(DMAm-co-GMA30) 80/20 *w*/*w*.

The first evidence on the success of the crosslinking reaction was obtained from the Attenuated Total Reflection Fourier Transform Infrared Spectroscopy (ATR-FTIR) characterization of membranes that were prepared by solution casting of the polymeric blends. As observed in Figure 3 for the blend P(VBCTMAM-co-AA20)/P(DMAm-co-GMA30) 80/20 *w*/*w*, the peak at 904 cm^−1^ which was attributed to the epoxy ring of GMA unit completely disappears after curing at 120 °C for one day.

### 2.4. Release Study of the Coatings

**Figure 4:**
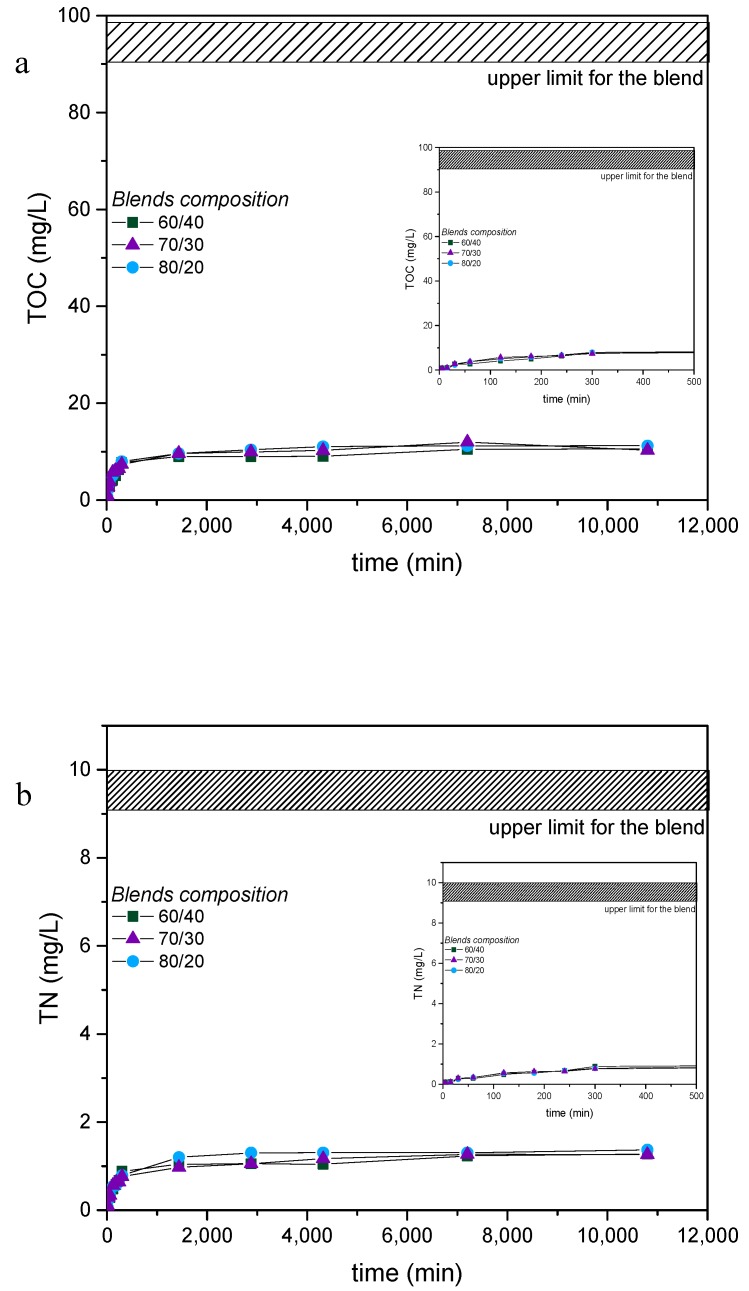
(**a**) Total carbon (TOC) and (**b**) Total nitrogen (TN) results of the 3.5%(*w/v*) NaCl solution used for the release study of the coated nets P(VBCTMAM-co-AA20)/P(DMAm-co-GMA30) 60/40, 70/30, 80/20 *w*/*w* for seven days.

The behavior of the coated nets in aqueous environment was investigated, prior to the fouling tests under accelerated fouling conditions [29]. Small pieces of the coated nets were immersed in pure water or 3.5%(*w*/*v*) NaCl solutions and the evolution of Total Organic Carbon (TOC) and Total Nitrogen (TN) was monitored with immersion time. Indicative results concerning the behavior in salt solutions of the coated nets cured at 120 °C are shown in Figure 4a,b. It is evident that the composition of the copolymers mixture used for coating hardly affects the behavior observed. In fact, for all three compositions, TOC and TN increase within the first few hours and then remain rather constant. These results suggest that a fraction of the polymeric material, consisted of either uncrosslinked copolymer chains or loosely crosslinked/grafted structures, is released under these conditions in the surrounding aqueous solution. The expected TOC and TN values if all material could be dissolved are indicated by the shadowed areas in Figure 4a,b respectively. It is clear that the fraction of the releasable material is low, about 10% of the whole material, suggesting that the two complementary copolymers were crosslinked to a large extent after the curing procedure at 120 °C. In contrast, we have verified that about half of the polymeric material is released when uncured coated nets are tested under the same conditions, indicating that the crosslinking reaction is not sufficiently effective and takes place to a much lower extent at room temperature. Finally, it should be noted that the presence of salt does not affect considerably the release behavior (Figures S1–S6), as expected, since neither of the complementary copolymers consists of ionic compounds that could be released to the NaCl solution through ion-exchange.

**Figure 5:**
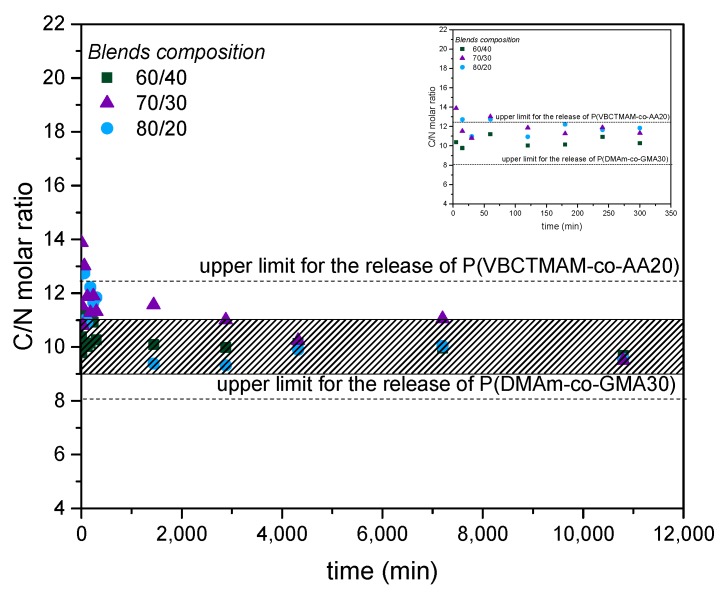
Evolution of the C/N molar ratio, determined from the TOC/TN measurements, after immersion of the coated nets P(VBCTMAM-co-AA20)/P(DMAm-co-GMA30) 60/40, 70/30, 80/20 *w*/*w* in 3.5%(*w*/*v*) NaCl solution for seven days.

The C/N molar ratios calculated from the TOC and TN values shown in Figure 4a,b are presented in Figure 5. The dotted lines in this Figure represent the C/N values for P(VBCTMAM-co-AA20) (upper line) and P(DMAm-co-GMA30) (lower line), as calculated from the chemical structures and also verified from the TOC and TN values determined using aqueous solutions of the two copolymers. The shadowed area represents the C/N molar ratios calculated for the three compositions of the polymer mixtures used for coating, if they were not crosslinked and were completely dissolved in the aqueous solution. As seen in Figure 5, most of the experimental data are very close to this area, indicating that the releasable material consists of both complementary copolymers, either as free chains or as graft structures. At the very beginning of the releasing process a tendency to higher values is observed, possibly indicating that P(VBCTMAM-co-AA20) chains are more readily dissolved initially. In any case, the most important finding of these results is that this small fraction of releasable material does contain the copolymer P(VBCTMAM-co-AA20). This is crucial since this copolymer is expected to present biocidal properties, leading possibly to a release-based antifouling action.

### 2.5. Antifouling Test in Accelerated Conditions

The blend with the highest content of cationic trimethylammonium compound P(VBCTMAM-co-AA20)/P(DMAm-co-GMA30) (80/20 *w*/*w*) was selected to measure its antifouling performance when treated on fishing nets. The coated net along with an uncoated (blank) net were immersed in glass tanks filled with seawater and remained for 14 days. In order to achieve accelerated conditions of algae growth, 3.5 mL of a Walne medium nutrient solution and 3.5 mL of algae aliquot were added into the seawater, while four multispectral lamps with a total of 500 lux of illuminance were placed over the tanks [30]. Concerning the algal growth cycle which lasts for six to seven days, the seawater-nutrient solution was renewed on the seventh day and the experiment was carried out for another week.

**Figure 6:**
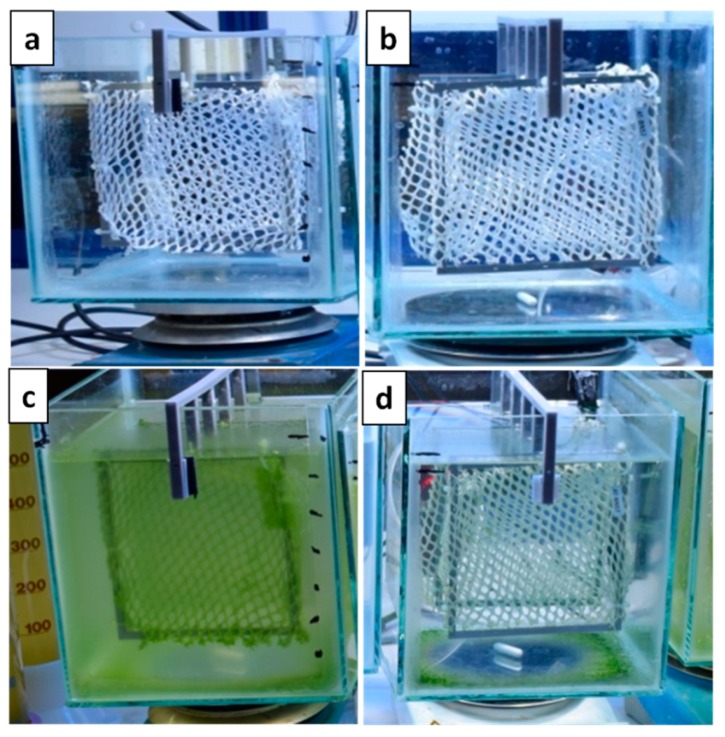
Photographs of the coated (**b**,**d**) and uncoated nets (**a**,**c**) after immersion in seawater solution the first day (**a**,**b**) and after seven days (**c**,**d**) in accelerated conditions.

Photographs of the immersed nets at the beginning of the experiment and after one week are presented in Figure 6. As may be seen there, the uncoated net (blank) had the highest fouling compared to the coated net. More specifically, enhanced algal growth is observed on the blank net accompanied by turbidity of the seawater solution. On the other hand, in the case of the coated net, even though there was a low algal growth observed at the bottom of the tank, the net itself exhibited good resistance for the seven day period.

The physicochemical parameters Salinity (S), pH, Dissolved Oxygen (DO), Turbitity (Tur) and Temperature (T) were measured for the whole period of the experiment in the tanks with the uncoated and coated nets (Table 1). There was no change in the salinity from the whole period of the experiment and also the turbidity of the seawater was not changed significant in the aquarium. The pH values show a gradual increase from 8.18 to 9.88 pH units and the dissolved oxygen reached the maximum value of 16.88 mg/L. Regarding the high pH values, it is expected that they will not affect the growth and production of algae during the experiment in a negative way, since the acceptable pH range for algae growth varies from pH seven to nine with the optimum range being from 8.2 to 8.7. The low temperature value 18.7 °C was observed at the beginning of the experiment and is the ambient seawater temperature, however temperature fluctuations are related with laboratory environmental conditions. In such temperature, light- and nutrient-conditioned algae growth is facilitated, photosynthesis is apparent and oxygen is produced [31,32].

The controlling factors for algae growth are light, nutrient availability, temperature, pH, salinity and the optimum range for these parameters applied during this study [31]. During photosynthesis, algae assimilates inorganic carbon and water, to produce organic matter and oxygen, using light as the driving source [31,32,33]. Our data indicate oxygen production during photosynthesis (Table 1), however the observed depletion of oxygen after 14 days in the reference and studied aquariums was attributed to the death phase of the algae cultures due to the production of toxic metabolites. Finally, the increase of the pH values can also be attributed to the phenomenon of photosynthesis [32].

**Figure 7:**
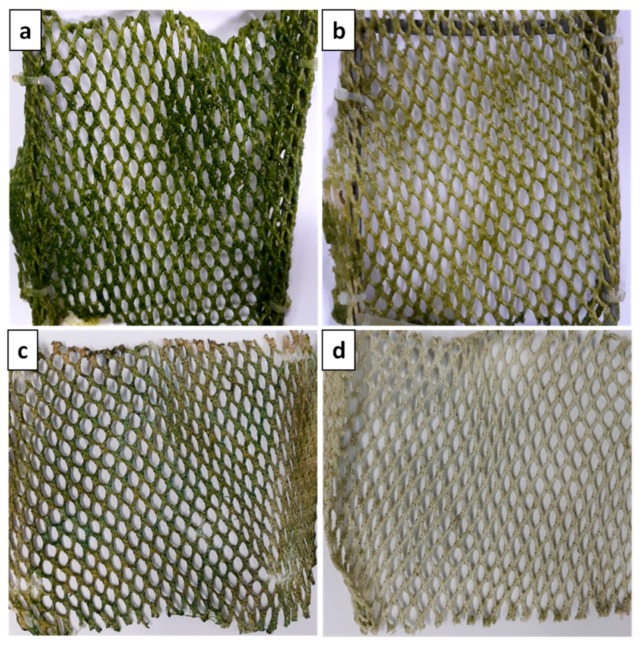
Photographs of the coated (**b**,**d**) and uncoated nets (**a**,**c**), before (**a**,**b**) and after (**c**,**d**) cleaning with water under high-pressure.

Nevertheless, right after the renewal of the seawater-nutrient solution, the settlement of fouling organisms was observed on the coated net, whereas the algal inhibition was significantly increased on the uncoated one (Figure 7a,b). This behavior shows that our coating does not completely prevent the fouling formation, but mainly delays the algal growth. Concerning the amount of fouling on the nets, there was not a feasible way to determine the mass of algae attached on the nets due to the co-existence of NaCl. Despite the short-term antifouling activity of the cationic polymeric coating P(VBCTMAM-co-AA20)/P(DMAm-co-GMA30) 80/20 *w*/*w*, it is important to evaluate the strength of the fouling adhesion on the coated net. For this reason, the coated and uncoated nets were cleaned under high-pressure water flow. As can be seen in Figure 7c,d, fouling was removed easily and more efficiently from the coated net than the uncoated one. Subsequently, these results lead to the potential use of this polymeric coating to improve the life expectancy of the aquaculture nets so that they can be used several times in the field after maintenance. It should be noted that the antifouling test of the polymeric coating P(VBCTMAM-co-AA20)/P(DMAm-co-GMA30) 80/20 *w*/*w*, as well as the cleaning of the nets were repeated once and showed the same results.

## 3. Experimental

### 3.1. Materials

The monomers N,N-dimethylacrylamide (DMAm), glycidyl methacrylate (GMA), acrylic acid (AA), 4-vinylbenzyl chloride (VBC), initiator azobisisobutyronitrile (AIBN), trimethylamine solution ~45% *(w/w*) in H_2_O (TMAM), as well as deuterium oxide (D_2_O) and deuterated chloroform (CDCl_3_) were purchased from Sigma-Aldrich (Steinheim, Germany) and used as received. The solvents N,N-dimethylformamide (DMF), chloroform (CHCl_3_), tetrahydrofuran (THF), hexane and acetone were purchased from Fischer (Pittsburgh, Pennsylvania, WI, USA) and used as received. Ultra-pure water was obtained by means of an SG apparatus water purification unit. Nylon nets (HelNet S.A., Schimatari, Greece) were used for coating.

### 3.2. Synthesis of Water-Soluble Copolymers

#### 3.2.1. Synthesis of P(DMAm-co-GMAx)

The copolymer poly(N,N-dimethylacrylamide-co-glycidyl methacrylate) was synthesized via free radical copolymerization of N,N-dimethylacrylamide (DMAm) and glycidyl methacrylate (GMA) monomers using AIBN as the initiator in THF at 70 °C. A typical polymerization reaction is as follows. A round bottom flask, equipped with a magnetic stirrer and a reflux condenser, was charged under argon atmosphere with 30 mL DMAm (0.293 mol), 16.7 mL GMA (0.126 mol) and 150 mL THF in order to acquire a concentration of 30% *w*/*v*. The initiator AIBN was then added 0.68g (4.1 mmol, 1% over the total monomer concentration). The solution was degassed and left under inert atmosphere, while vigorously stirred in an oil bath at 70 °C for 2 days and then was concentrated in the rotary evaporator. The product was received by precipitation in hexane, filtered, washed with hexane and dried in a vacuum oven at 45 °C for 24 h. The mole fraction (x) of the GMA unit in the copolymer P(DMAm-co-GMAx) was determined by ^1^H NMR characterization in CDCl_3_. Moreover, the molecular weight of the copolymer was calculated M_w_: 7000 g/mol through size exclusion chromatography in chloroform.

#### 3.2.2. Synthesis of P(VBCTMAM-co-AAx)

The precursor copolymer poly(4-vinylbenzyl chloride-co-acrylic acid) was synthesized via free radical copolymerization of vinyl benzyl chloride (VBC) and acrylic acid (AA) monomers using AIBN as the initiator in DMF at 80 °C. The mole fraction (x) of the AA unit in the copolymer P(VBC-co-AAx) was determined by ^1^H NMR characterization in CDCl_3_ and the molecular weight determined through size exclusion chromatography in chloroform was found M_w_: 15,000 g/mol. The experimental procedure for the synthesis and characterization of copolymer was described previously [25,34]. The synthesized copolymer is further modified with trimethylamine as follows. In a round bottom flask, equipped with a magnetic stirrer, 10.0 g of copolymer P(VBC-co-AA20) (73.3 mmol, 1 eq) was first dispersed in 200 mL of H_2_O and then 12.3 mL of trimethylamine (TMAM) (80.6 mmol, 1.1 eq) was added to the solution. The reaction mixture was left at room temperature for 1 day and the product was obtained through drying at room temperature for 1 day.

### 3.3. Preparation of Antifouling Coatings on Nets

For the preparation of the water-soluble blends, the copolymers P(VBCTMAM-co-AA20) and P(DMAm-co-GMA30) were dissolved in H_2_O at a 4% (*w*/*v*) and 10% (*w*/*v*) concentration respectively. The mother solutions of complementary copolymers were mixed at compositions 60/40, 70/30, 80/20 *w*/*w* and left overnight under mild stirring at room temperature. Afterwards, pre-weighed nets (15 × 15 cm) were immersed in the blend solutions for a while and then left at room temperature for 24 h to dry. The polymer uptake of the nets was 20–25% (*w*/*w*). The coated nets were subsequently cured at 120 °C for one day. Uncured nets were also used for comparison reasons.

### 3.4. Preparation of Samples for Total Organic Carbon (TOC) and Total Nitrogen (TN) Measurements

0.2 g of the coated nets were immersed in pure water or 3.5%(*w*/*v*) NaCl solutions. At several time intervals, 1mL aliquots were withdrawn, diluted to 10 mL with pure water or 3.5%(*w*/*v*) NaCl solution and the TOC/TN content was measured. The volume of the aliquot was immediately replaced, in order to maintain the total volume constant.

### 3.5. Antifouling Tests in Accelerated Conditions

The coated nets as well as uncoated (blank) nets were placed into glass tanks filled with sea water, 3.5 mL of a Walne medium nutrient solution and 3.5 mL of algae aliquot in order to achieve 1.0 mL of algae-nutrient mixture per litter of sea water. The tanks were placed under 4 multispectral lamps with a total of 500 lux of illuminance. Experimental conditions consist of sea water temperature ranged from 18.4–29.7 °C, pH from 8.17 to 9.88, O_2_ from 5.63 to 16.88 ppm and salinity from 39.7 to 41.0 g/kg. The accelerating eutrophication conditions were daily optically examined in the blank (uncoated net), ensuring that adequate repeatable conditions were obtained the fourth day of each experiment inception.

### 3.6. Characterization Techniques

#### 3.6.1. Proton Nuclear Magnetic Resonance (^1^H NMR)

The ^1^H NMR spectra were obtained at 400 MHz at 300 K on a Bruker AVANCE DPX 400 spectrometer (Bruker BioSpin GmbH, Magnet Division, Karlsruhe, Germany). The ^1^H NMR spectra were used to determine the chemical composition of the copolymers.

#### 3.6.2. Attenuated Total Reflection Fourier Transform Infrared Spectroscopy (ATR-FTIR)

The ATR-FTIR spectra of the copolymers P(VBC-co-AA20) and P(DMAm-co-GMA30), the quaternized copolymer P(VBCTMAM-co-AA20), and the cross-linked blends were recorded using Bruker Optic’s Alpha-P Diamond ATR Spectrometer of Bruker Optics GmbH (Ettlingen, Germany).

#### 3.6.3. Size Exclusion Chromatography (SEC)

A Polymer Lab chromatographer (Agilant Technologies, Santa Clara, CA, USA) was used for the determination of copolymers’ P(VBC-co-AAx) and P(DMAm-co-GMAx) molecular weights. The measurements were carried out at 25 °C by using a Marathon II HPLC pump, a Fasma 500 UV/vis detector, and two PLgel 5 μm mixed columns. Chloroform (CHCl_3_) was used as the mobile phase, polystyrene standards were used for calibration, and the software Clarity v.3.0.07.662 was used for the spectra analysis.

#### 3.6.4. Scanning Electron Microscopy (SEM)

Scanning electron microscopy of cross-linked blends (SEM, JEOL 6300, Tokyo, Japan, instrument equipped with an X-ray Energy Dispersive Spectrometer, EDS) was performed to investigate their morphologies. The samples were sputtered with gold to produce electric conductivity before SEM examination.

#### 3.6.5. Total Organic Carbon (TOC) and Total Nitrogen (TN) Measurements

Simultaneous analyses of TOC and TN were carried out using a Schimadzu TOC analyzer (TOC-VCSH) coupled to a chemiluminescence detector (TNM-1 TN unit) [35].

## 4. Conclusions

This work demonstrates a simple and scalable fabrication approach for the development of hydrophilic, positively charged stable surfaces from water-soluble polymers as potential antifouling coatings for aquaculture nets. Stable coatings were developed after optimization of the blend composition and the treatment procedure. The release rate of the coated nets was studied in selected simulants and showed that the fraction of the releasable material is low, about 10% (*w/w)* of the whole material, suggesting that the two complementary copolymers were crosslinked to a large extent after the curing procedure. An evaluation of the antifouling activity of the coated nets was performed in tanks with seawater-nutrient solutions under accelerated conditions for a period of 14 days with a renewal of the seawater solution between the two weeks. The coated net in contrast with the uncoated one exhibited high resistance to biofouling adhesion during the first period, though after the renewal of seawater in the tank, algae settlement on the coated net was observed. However, when cleaned with high-pressure water, the fouling was removed more easily and efficiently from the coated net rather than the uncoated one. In conclusion, this cationic coating appears to provide a dual function: it acts as a biocidal against marine microorganisms for a specific time period as well as a protective surface against the biofilm adhesion on the nets. Such knowledge is a prerequisite in order to optimize the efficacy and duration of the antifouling action of this novel polymeric coating’s potential for aquaculture applications.

## Figures and Tables

**Table 1:** Measured physicochemical parameters in the tanks with the uncoated (uc) and coated (c) nets.

Days	Salinity (%)		pH		Turbitity (FAU)		Dissolved Oxygen (mg/l)		Temperature (°C)	
	uc	c	uc	c	uc	c	uc	c	uc	c
1	39.5	39.7	8.17	8.18	10.3	10.5	7.80	7.93	18.4	18.7
3	41.0	41.0	8.39	8.34	10.9	10.3	8.06	5.63	24.4	25.8
7	41.0	41.0	10.31	9.33	22.7	10.8	15.58	16.88	27.1	27.3
14	41.0	41.0	9.83	9.88	68.6	10.9	12.07	15.86	28.9	29.7

## Supplementary Materials

The following are available online. Figure S1: (a) Total carbon (TOC) and (b) Total nitrogen (TN) results of the 3.5% (*w*/*v*) NaCl solution and water used for the release study of the coated nets P(VBCTMAM-co-AA20)/P(DMAm-co-GMA30) 60/40 *w/w* for seven days, Figure S2: Evolution of the C/N molar ratio, determined from the TOC/TN measurements, after immersion of the coated nets P(VBCTMAM-co-AA20)/P(DMAm-co-GMA30) 60/40 *w/w* in 3.5% (*w*/*v*) NaCl solution and water for seven days, Figure S3: (a) Total carbon (TOC) and (b) Total nitrogen (TN) results of the 3.5% (*w*/*v*) NaCl solution and water used for the release study of the coated nets P(VBCTMAM-co-AA20)/P(DMAm-co-GMA30) 70/30 *w/w* for seven days, Figure S4: Evolution of the C/N molar ratio, determined from the TOC/TN measurements, after immersion of the coated nets P(VBCTMAM-co-AA20)/P(DMAm-co-GMA30) 70/30 *w/w* in 3.5% (*w*/*v*) NaCl solution and water for seven days, Figure S5: (a) Total carbon (TOC) and (b) Total nitrogen (TN) results of the 3.5% (*w*/*v*) NaCl solution and water used for the release study of the coated nets P(VBCTMAM-co-AA20)/P(DMAm-co-GMA30) 80/20 *w/w* for seven days, Figure S6: Evolution of the C/N molar ratio, determined from the TOC/TN measurements, after immersion of the coated nets P(VBCTMAM-co-AA20)/P(DMAm-co-GMA30) 80/20 *w/w* in 3.5% (*w*/*v*) NaCl solution and water for seven days.
