# Supplementary material for: Polymeric Coatings Based on Water-Soluble Trimethylammonium Copolymers for Antifouling Applications
Source: Molecules. 2020 Apr 6;25(7):1678. doi: 10.3390/molecules25071678 (PMC7180454; doi:10.3390/molecules25071678)
Supplement: Supplementary file 1 [file molecules-25-01678-s001.pdf]

Supplementary Material

Polymeric coatings based on water-soluble trimethylammonium copolymers for antifouling applications

Artemis Tsagdi, Denisa Druvari, Dionisios Panagiotaras, Pavlos Avramidis, Vlasoula Bekiari and Joannis K. Kallitsis

Contents

Figure S1: (a) Total carbon (TOC) and (b) Total nitrogen (TN) results of the 3.5% (*w/v*) NaCl solution and water used for the release study of the coated nets P(VBCTMAM-co-AA20)/P(DMAm-co-GMA30) 60/40 *w/w* for seven days.

Figure S2: Evolution of the C/N molar ratio, determined from the TOC/TN measurements, after immersion of the coated nets P(VBCTMAM-co-AA20)/P(DMAm-co-GMA30) 60/40 *w/w* in 3.5% (*w/v*) NaCl solution and water for seven days.

Figure S3: (a) Total carbon (TOC) and (b) Total nitrogen (TN) results of the 3.5% (*w/v*) NaCl solution and water used for the release study of the coated nets P(VBCTMAM-co-AA20)/P(DMAm-co-GMA30) 70/30 *w/w* for seven days.

Figure S4: Evolution of the C/N molar ratio, determined from the TOC/TN measurements, after immersion of the coated nets P(VBCTMAM-co-AA20)/P(DMAm-co-GMA30) 70/30 *w/w* in 3.5% (*w/v*) NaCl solution and water for seven days.

Figure S5: (a) Total carbon (TOC) and (b) Total nitrogen (TN) results of the 3.5% (*w/v*) NaCl solution and water used for the release study of the coated nets P(VBCTMAM-co-AA20)/P(DMAm-co-GMA30) 80/20 *w/w* for seven days.

Figure S6: Evolution of the C/N molar ratio, determined from the TOC/TN measurements, after immersion of the coated nets P(VBCTMAM-co-AA20)/P(DMAm-co-GMA30) 80/20 *w/w* in 3.5% (*w/v*) NaCl solution and water for seven days.

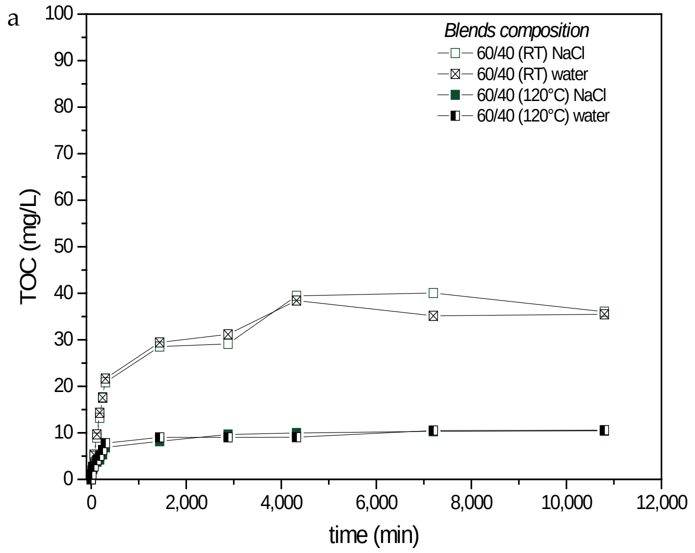

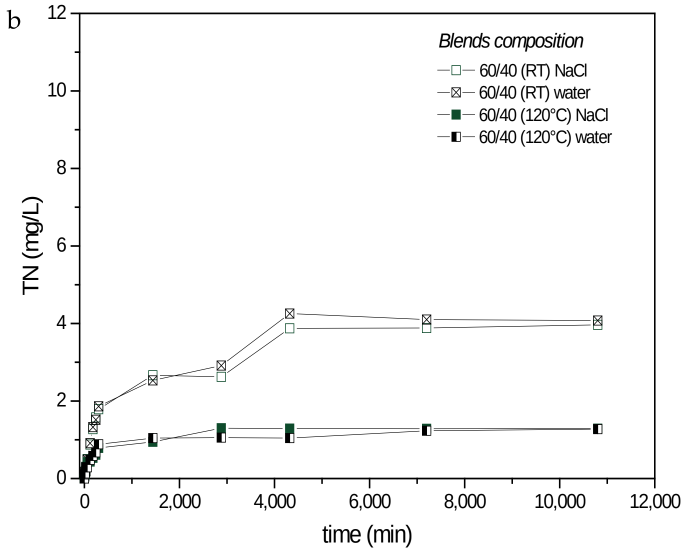

Figure S1. (a) Total carbon (TOC) and (b) Total nitrogen (TN) results of the 3.5% (*w*/*v*) NaCl solution and water used for the release study of the coated nets P(VBCTMAM-co-AA20)/P(DMAm-co-GMA30) 60/40 *w*/*w* for seven days.

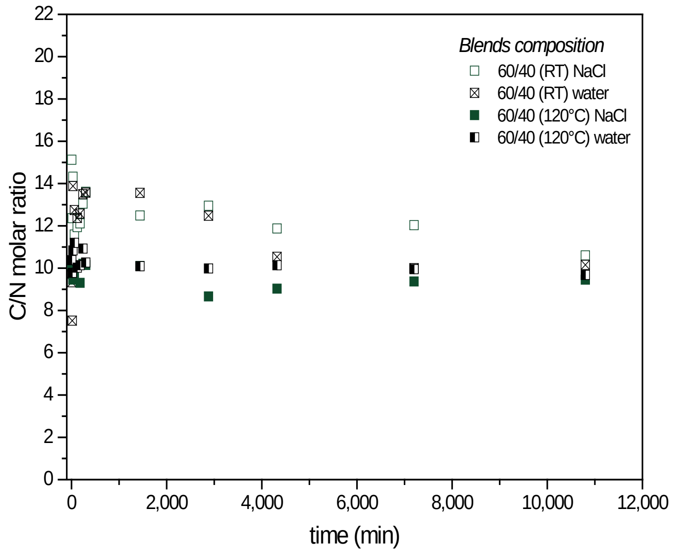

Figure S2. Evolution of the C/N molar ratio, determined from the TOC/TN measurements, after immersion of the coated nets P(VBCTMAM-co-AA20)/P(DMAm-co-GMA30) 60/40 *w/w* in 3.5% (*w/v*) NaCl solution and water for seven days.

a

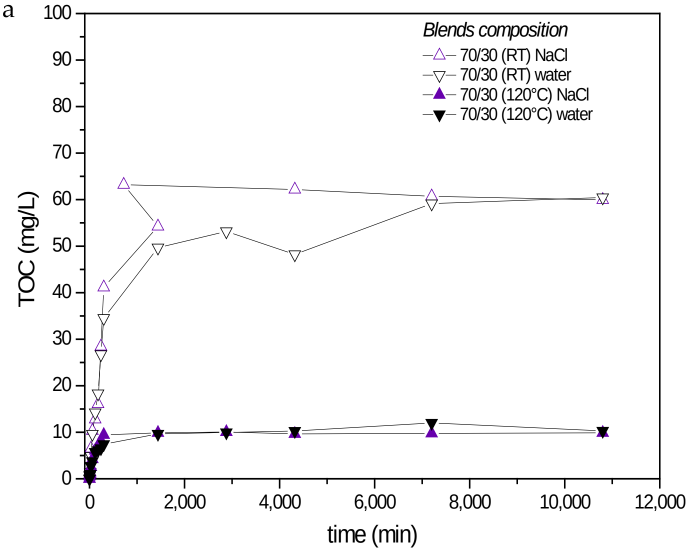

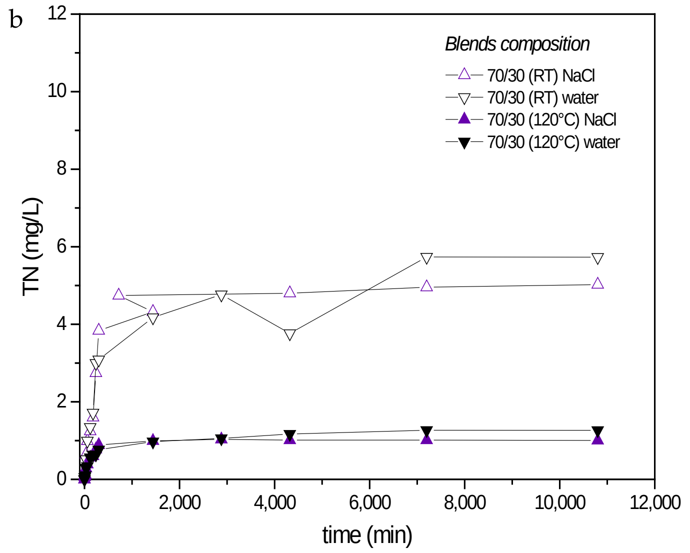

Figure S3. (a) Total carbon (TOC) and (b) Total nitrogen (TN) results of the 3.5% (*w*/*v*) NaCl solution and water used for the release study of the coated nets P(VBCTMAM-co-AA20)/P(DMAm-co-GMA30) 70/30 *w*/*w* for seven days.

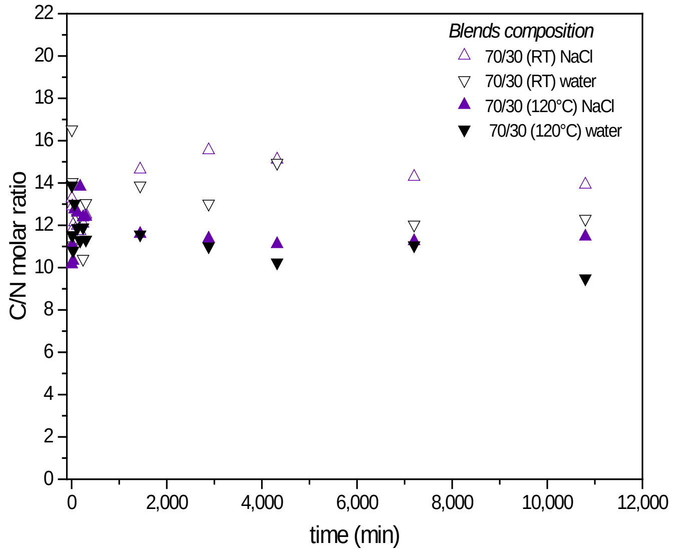

Figure S4. Evolution of the C/N molar ratio, determined from the TOC/TN measurements, after immersion of the coated nets P(VBCTMAM-co-AA20)/P(DMAm-co-GMA30) 70/30 *w*/*w* in 3.5% (*w*/*v*) NaCl solution and water for seven days.

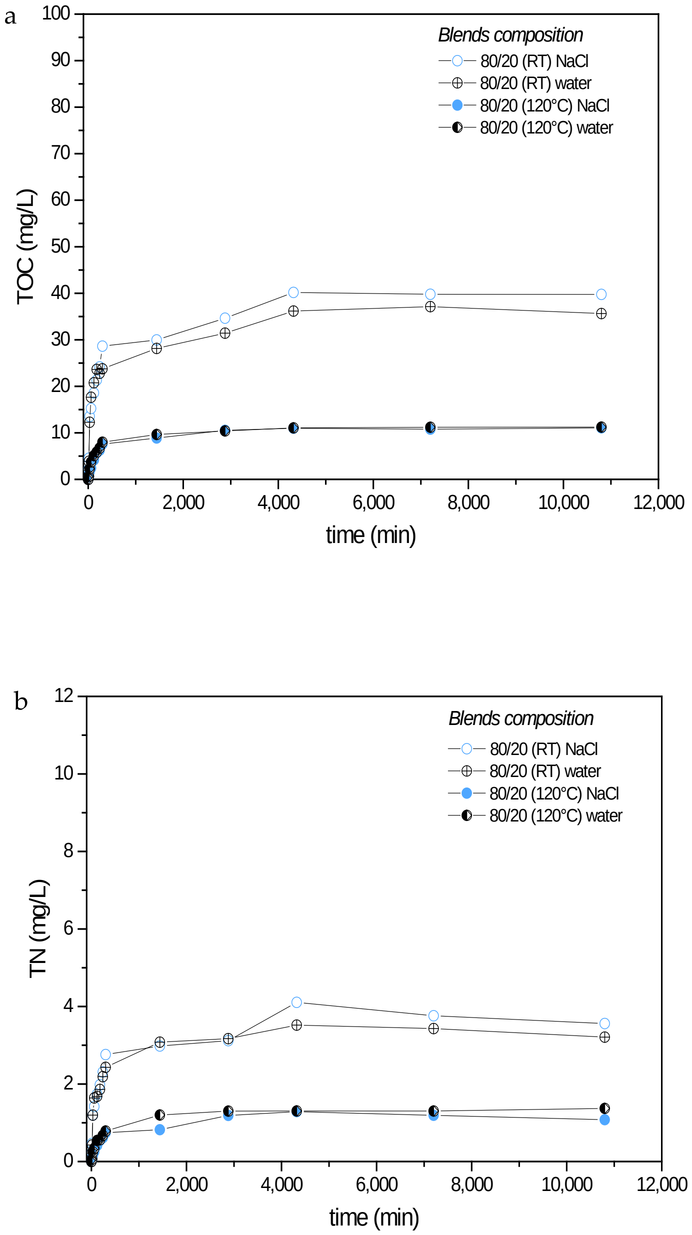

Figure S5. (a) Total carbon (TOC) and (b) Total nitrogen (TN) results of the 3.5% (*w*/*v*) NaCl solution and water used for the release study of the coated nets P(VBCTMAM-co-AA20)/P(DMAm-co-GMA30) 80/20 *w*/*w* for seven days.

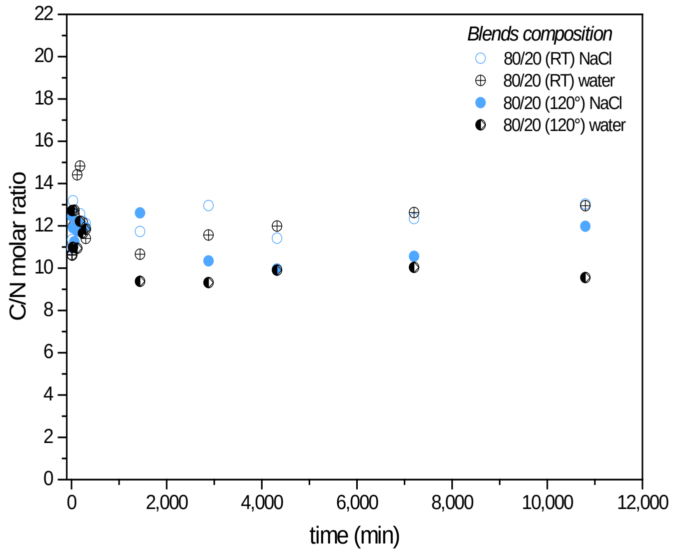

Figure S6. Evolution of the C/N molar ratio, determined from the TOC/TN measurements, after immersion of the coated nets P(VBCTMAM-co-AA20)/P(DMAm-co-GMA30) 80/20 *w/w* in 3.5% (*w/v*) NaCl solution and water for seven days.
